# Supplementary material for: Dynamics analysis of disturbance propagation in ecosystem with proportional migration based on epidemic model
Source: PLoS One. 2026 Jun 1;21(6):e0344916. doi: 10.1371/journal.pone.0344916 (PMC13225436; doi:10.1371/journal.pone.0344916)
Supplement: Attachment — Submitted filename: Reviewer Attachments for Manuscript Number PONE-D-25-58468.pdf [file pone.0344916.s001.pdf]

The manuscript ingeniously analogizes the propagation dynamics of ecological disturbances in food webs to the spread of infectious diseases on complex networks. It constructs a disturbance propagation model for ecological networks, analyzes and verifies the propagation process. The research idea is novel, the logic is clear, and the conclusions have practical reference value.

The manuscript is well-written, with model derivation and empirical analysis supporting the core viewpoints, meeting the basic requirements for publication. It is recommended to be accepted after minor revisions.

Major Comments:

1. In the abstract, the definition of " medium-sized and large-sized " in the phrase " the medium-sized and large-sized neighbor nodes" is somewhat ambiguous. Does this refer to the degree (number of connections) of the nodes, their centrality, betweenness centrality, or other measures related to biomass/size?.
2. The introduction provides a good overview of the application of complex network theory in ecosystems and the research foundation of infectious disease models. However, it should be supplemented with a clearer statement of the core differences and innovative aspects of this study compared to existing literature. It is recommended to explicitly highlight the unique contributions

of this work in terms of model assumptions (e.g., definition of species states, disturbance propagation mechanisms), research objectives (e.g., optimization of protection strategies), or empirical context (validation using real food web data), to avoid ambiguity regarding its distinction from prior research.

3. It is advisable to briefly elaborate on why the food chain can be considered the core pathway for disturbance propagation (e.g., based on the logic of energy flow and resource dependence among species), as well as the rationale behind the "immunity" analogy (e.g., how protective measures equivalently reduce the probability of species being disturbed or enhance their recovery capacity). This would strengthen the theoretical foundation of the model construction.
4. While the manuscript outlines the research framework (model construction, strategy comparison, empirical validation), the introduction lacks sufficient context for the definition of key parameters. It is suggested to briefly mention the central role of core parameters (such as disturbance propagation probability, species recovery probability, and the basic reproduction number) either at the end of the introduction or in a preliminary section of the research methods, to ensure smoother logical transition to the model derivation in subsequent sections.

5. Standardize the literature citation format throughout the manuscript to ensure academic rigor and consistency in presentation..
