# Supplementary material for: Dynamics analysis of disturbance propagation in ecosystem with proportional migration based on epidemic model
Source: PLoS One. 2026 Jun 1;21(6):e0344916. doi: 10.1371/journal.pone.0344916 (PMC13225436; doi:10.1371/journal.pone.0344916)
Supplement: Attachment — Submitted filename: Response.pdf [file pone.0344916.s002.pdf]

1. **Model Assumptions and Ecological Realism:**

   - The model assumes that disturbances spread symmetrically through predator-prey links. Could the authors discuss the ecological justification for this assumption, especially given that disturbances (e.g., pollution, habitat loss) may affect species asymmetrically?
   - How does the model account for the strength of interactions (e.g., interaction strength, energy flow), which may influence disturbance propagation more than topology alone?

2. **Parameter Estimation and Sensitivity:**

   - The parameters $\beta, \sigma, \delta, \mu, \gamma, b$ are central to the model. Were these parameters estimated from ecological data or chosen for theoretical analysis? If the latter, how sensitive are the main results to these choices?
   - Is the value $a = 1$ in $\varphi(k) = ak$ justified empirically, or is it a simplifying assumption? How might a more general form affect the basic reproduction number ρ?

3. **Immunization Strategies and Practical Implementation:**

   - The paper concludes that active immunization (protecting neighbors of disturbed species) is most effective. How would this strategy be implemented in real ecosystems, where the state of species (disturbed/recovered) may not be directly observable?
   - Are there ecological or economic constraints that might limit the feasibility of targeted or active immunization in conservation practice?

4. **Numerical Simulations and Model Validation:**

   - The simulations use a fixed initial condition $I_k(0) = 0.1$. How do the results change with different initial conditions, especially for sparse or localized disturbances?
   - The degree distribution is fitted as $P(k) = 0.26/k$. Was this fit validated against other possible distributions (e.g., power-law, exponential)? How robust are the conclusions to the degree distribution form?

5. **Global Stability Proofs:**

   - The proofs of global stability for both disease-free and endemic equilibria are highly technical. Could the authors provide more intuitive explanations or biological interpretations of these stability conditions?
   - In Theorem 5, the proof of global stability for the endemic equilibrium relies on a complex sequence construction. Is there a more straightforward Lyapunov function or method that could be applied?

Minor Comments

1. The manuscript contains several typographical and formatting issues, especially in the mathematical notation (e.g., inconsistent use of brackets, alignment in equations). A thorough proofreading is recommended.

2. Some references (e.g., [37], [38], [41]) are cited in the text but do not appear in the reference list. Please ensure all citations are complete and consistent.

3. The figures (e.g., Fig. 2, Fig. 3) are referenced but not included in the provided text. Please confirm that all figures and tables are properly labeled and referenced.

This manuscript presents a novel and mathematically rigorous approach to modeling disturbance propagation in ecosystems using epidemic models. The theoretical results are well-supported, and the application to a real food web adds practical relevance. However, further clarification on ecological realism, parameter choices, and practical implementation of immunization strategies would strengthen the paper.

I look forward to the authors' responses.
