# Supplementary material for: Dynamics analysis of disturbance propagation in ecosystem with proportional migration based on epidemic model
Source: PLoS One. 2026 Jun 1;21(6):e0344916. doi: 10.1371/journal.pone.0344916 (PMC13225436; doi:10.1371/journal.pone.0344916)
Supplement: Attachment — Submitted filename: Review Report-PONE-D-25-58468.pdf [file pone.0344916.s003.pdf]

Review Report

Manuscript ID: PONE-D-25-58468

This paper develops a novel mathematical framework to model the propagation of ecological disturbances by adapting a SIRS epidemic model to complex networks. The authors abstract species as nodes and predator–prey relationships as edges in a food web, where disturbances can spread from initially affected species to their neighbors. They derive a key epidemiological threshold, the basic reproduction number (R_0), proving that the disturbance will die out if $R_0 < 1$ and become endemic if $R_0 > 1$, with rigorous global stability analysis for both equilibria. Using real data from a New Zealand pine forest food web, they validate the model numerically and further analyze species conservation strategies, concluding that proactively protecting the highly connected neighbors of disturbed species is the most effective intervention to suppress disturbance spread.

The following observations must be cleared up before it is considered for publication:

1. The introduction should make a compelling case for why the study is useful along with a clear statement of its novelty or originality by providing relevant information and providing answers to basic questions such as:
   (a) What is already known in the literature?
   (b) What was done and how was it done?

2. The model assumes disturbance propagates with a probability ρ along food web edges. Given that ecological interactions vary in strength and type (e.g., strong/weak predation, competition), how does the assumption of a uniform propagation probability β impact the model's biological realism? Could the model be extended to include weighted edges based on interaction strength?

3. The expression for ρ (Eq. 2.2.12) is crucial. Clarify the step-by-step derivation from the next-generation matrix method or the local stability analysis of the disease-free equilibrium. The current derivation in Lemma 3 seems to appear from a self-consistency condition; a more standard epidemiological derivation would strengthen the manuscript.

4. The proofs for the global stability of both the disease-free (Theorem 2) and endemic (Theorem 5) equilibria are highly technical and rely on constructing complex Lyapunov functions and sequences. For the sake of reproducibility and clarity, provide more detailed intermediate steps or intuition behind the construction of these functions, particularly in Theorem 5.

5. The degree distribution for the Otago food web is fitted to a power-law $P(k) = 0.26/k$ (Page 28). What statistical tests were performed to justify this specific functional form over alternatives (e.g., exponential, log-normal)? Furthermore, how were the key parameters $(\beta, \sigma, \delta, \mu, \gamma, b)$ assigned specific numerical values for the simulations, and were these values informed by empirical data or chosen arbitrarily for illustration?

6. In Figures 5–9, the results are presented for a node of degree $k = 5$. How were the initial conditions $S_k(0), I_k(0), R_k(0)$ set for these simulations, especially for the endemic case ($\rho > 1$)? Specify if the results are for a single node, an average over all nodes of degree $k = 5$, or a density across the network.

7. Figures 7–9 show the system's state evolving over time for different parameters. To assess the robustness of the conclusions, have the authors performed a sensitivity analysis on the initial conditions or the network structure itself? For instance, how do the stability results change if the network is randomized while preserving the degree distribution?

8. In Section 3, three immunization strategies are compared based on their resulting R_0 ($\rho_3 < \rho_2 < \rho_1$). The claim that active immunization is "most effective" relies on this inequality. However, the comparison assumes the same "cost" (i.e., the same number of protected species, $\bar{\varphi}$). Provide a more explicit comparison, perhaps with a simulation, showing the final outbreak size $I(t)$ for each strategy when the same total number of species is protected.

9. The model employs the "empty-lattice" theory to handle network dynamics (species migration/extinction). How does this assumption of immediate replacement by an identical, susceptible node affect the long-term dynamics, especially regarding the conservation of keystone species whose loss might fundamentally alter the network topology, not just a node's state?

10. Figure 2 is described as a topology map but is not visually present in the provided text. If included, does it provide any topological analysis (e.g., highlighting hubs, clustering coefficient, community structure)? A simple visualization of 85 nodes is often a "hairball." How was this figure rendered to provide insight into the network's structure that informs the dynamical results?

11. The simulations in Figures 5 and 6 are used to validate the theoretical threshold $\rho_c = 1$. Do the authors observe a sharp transition in the final density of disturbed species $I(\infty)$ as β crosses the critical value β_c predicted by Eq. (2.2.13)? A figure plotting $I(\infty)$ against β (or ρ) would provide stronger numerical evidence for the existence of this epidemic threshold.

12. Authors may look for some punctuation, typos and editing issues.
