# Supplementary material for: Dynamics analysis of disturbance propagation in ecosystem with proportional migration based on epidemic model
Source: PLoS One. 2026 Jun 1;21(6):e0344916. doi: 10.1371/journal.pone.0344916 (PMC13225436; doi:10.1371/journal.pone.0344916)
Supplement: Attachment — Submitted filename: Response to Reviewers.docx [file pone.0344916.s005.docx]

Dear editor,

Thank you for your useful comments and suggestions on our manuscript. We have modified the manuscript accordingly.

**Reviewer #1:**

**Q1**: The model assumes that disturbances spread symmetrically through predator-prey links. Could the authors discuss the ecological justification for this assumption, especially given that disturbances (e.g., pollution, habitat loss) may affect species asymmetrically?

**A1**: We sincerely thank the reviewer for this insightful comment. We agree that in real-world ecosystems, specific disturbances often exhibit directional bias. For instance, pollutants typically accumulate upwards through biomagnification, while habitat loss affecting top predators cascades downwards to lower trophic levels.

However, in our proposed model, "disturbance" is abstracted as a state of ecological instability rather than a specific physical flow of matter. Our assumption of symmetric propagation through predator-prey links is based on the ecological principle of mutual dependency between interacting species: If a prey species is disturbed, its predators are destabilized due to food scarcity. If a predator species is disturbed, its prey is destabilized due to the release from predation pressure, potentially leading to population outbreaks or collapse. Therefore, while the mechanistic causes differ, the propagation of instability can occur in both directions along a food web link. Our model employs this simplification to capture the generalized dynamics of how perturbations spread through the network topology.

To address your concern, we have added a paragraph in Section 2.1 to explicitly discuss this assumption, acknowledge the asymmetry of specific disturbances, and justify the bidirectional approach used in our theoretical framework.

**Q2**: How does the model account for the strength of interactions (e.g., interaction strength, energy flow), which may influence disturbance propagation more than topology alone?

**A2**: We appreciate the reviewer’s valuable observation regarding the role of interaction strength. We acknowledge that in real-world ecosystems, the magnitude of energy flow and interaction strength varies significantly across links, which can strongly influence dynamical stability.In the current version of our model, we adopted an unweighted network approach to maintain mathematical tractability. Specifically:Simplification for Analytical Solutions: Our primary goal was to derive an analytical expression for the basic reproduction number ($\rho$) and to establish the global stability conditions for the disease-free and endemic equilibria. Introducing heterogeneous interaction weights at this stage would significantly complicate the derivation of these threshold conditions.Average Probability: The parameter $\beta$ in our model can be interpreted as an average interaction strength across the ecosystem. While it does not capture the variance of specific links, it provides a baseline for understanding how the overall connectivity facilitates disturbance spread.However, we fully agree that incorporating interaction heterogeneity is a crucial next step. Strong interactions may act as highways for disturbance, while weak interactions might dampen it. We have updated the Discussion section to explicitly state this limitation and outline the integration of weighted networks and energy flow data as a priority for our future research.

**Q3**: The parameters $\beta, \sigma, \delta, \mu, \gamma, b$ are central to the model. Were these parameters estimated from ecological data or chosen for theoretical analysis? If the latter, how sensitive are the main results to these choices?

**A3:** We wish to clarify that the dynamical parameters $\beta, \sigma, \delta, \mu, \gamma, b$ used in the numerical simulations were chosen for theoretical analysis to illustrate the qualitative behavior of the model. However, we have rigorously assessed the sensitivity of our main results to these parameter choices in two ways:

1. Analytical Sensitivity: Our theoretical derivation of the basic reproduction number, $\rho$ (Eq. 2.2.12 ), provides an exact analytical expression of how the disturbance threshold depends on these parameters.
2. Numerical Sensitivity: In Section 4, we conducted extensive numerical simulations to examine how variations in these parameters affect the system dynamics.

These simulations confirm that while the specific numerical value of the endemic equilibrium ($I^{*}$) changes with these parameters, the qualitative stability of the system remains robust and is strictly determined by whether the parameter combination satisfies the threshold condition$\rho>1$ or $\rho<1$.

To prevent confusion, we have added a clarification statement in Section 4 regarding the choice of these parameters.

**Q4**: Is the value $a=1$ in $\phi(k)=ak$ justified empirically, or is it a simplifying assumption? How might a more general form affect the basic reproduction number $\rho$?

**A4**: We thank the reviewer for this question. The choice $a=1$in $\phi(k)=ak$ is a simplifying assumption, rather than an empirically fitted value. This linear form is adopted as a first-order approximation to capture the dependence of disturbance contribution on node degree while maintaining analytical tractability and emphasizing structural effects.

If a more general form is considered, the basic reproduction number$\rho$ would be modified through a corresponding scaling or weighting of degree-dependent terms. However, the overall threshold structure of 𝜌 and the qualitative conclusions regarding disturbance persistence and extinction remain unchanged.

We have added a remark in Section 2.1 to clarify this simplification and its linear effect on the threshold.

**Q5**: The paper concludes that active immunization (protecting neighbors of disturbed species) is most effective. How would this strategy be implemented in real ecosystems, where the state of species (disturbed/recovered) may not be directly observable?

**A5**: We appreciate the reviewer raising this critical point regarding practical implementation. We acknowledge that "Active Immunization" relies on dynamic state information, which is indeed challenging to observe continuously in large-scale ecosystems.

To bridge the gap between our theoretical conclusion and real-world application, we propose a two-tier implementation strategy:

Use of Sentinel Species: In practice, conservationists often monitor specific "indicator" or "sentinel" species rather than the entire food web. These species are selected for their sensitivity to environmental stress and ease of observation. Under our "Active Immunization" framework, if a sentinel species is observed to be disturbed, protective resources should be immediately allocated to its direct trophic neighbors.

Fallback to Targeted Protection: In scenarios where real-time observation is entirely impossible, the strategy can operate based on static topology. Our results indicate that "Active Immunization" prioritizes protecting the medium and large-degree neighbors of disturbed nodes. In scale-free ecological networks, high-degree species are statistically the most frequent neighbors of any random node. Therefore, proactively protecting known Keystone Species serves as a robust, static proxy for active immunization when dynamic state information is unavailable.

We have added a "Practical Implementation" remark at the end of Section 3.3 to explicitly discuss these real-world operational strategies.

**Q6**: Are there ecological or economic constraints that might limit the feasibility of targeted or active immunization in conservation practice?

**A6**: We thank the reviewer for highlighting the practical constraints of conservation, which are indeed critical context for our theoretical results. While our model treats the protection of any node as mathematically equivalent, the economic costs in reality are highly heterogeneous.

Despite these constraints, our findings serve as a crucial component of a cost-benefit analysis. By quantifying the theoretical "benefit", our model suggests that despite the high economic "cost" of protecting hub species or neighbors of disturbed nodes, the return on investment—in terms of preventing total network collapse—is maximized by these strategies.

We have expanded the Conclusion (Section 5) to explicitly acknowledge these real-world constraints.

**Q7**: The simulations use a fixed initial condition $I_{k}(0)=0.1$. How do the results change with different initial conditions, especially for sparse or localized disturbances?

**A7**: We thank the reviewer for this question regarding the system's sensitivity to initial conditions. Our response is grounded in the rigorous mathematical proofs provided in Section 2.3. We have proven that the equilibrium points of our model are globally asymptotically stable. Mathematically, this means that the "basin of attraction" for these equilibria encompasses the entire feasible phase space (where $I(0)>0$). Therefore, the final steady state of the ecosystem is independent of the initial scale of the disturbance. The value $I_{k}(0)=0.1$ was selected in our simulations primarily to visualize the convergence process clearly within a reasonable time window.

We have added a clarifying statement in Section 4 to explicitly state that the results are robust to variations in initial conditions due to the proven global stability.

**Q8**: The degree distribution is fitted as $P(k)=0.26/k$. Was this fit validated against other possible distributions (e.g., power-law, exponential)? How robust are the conclusions to the degree distribution form?

**A8**: We appreciate the reviewer’s insightful comment regarding the network topology.Regarding the choice of the distribution function, the fit $P(k)=0.26/k$ was selected based on the empirical data from the Otago pine forest food web. As shown in Fig. 4 of the original manuscript, this function yielded a high coefficient of determination ($R^{2}=0.9024$), indicating it captures the topological characteristics of this specific ecosystem accurately. While we did not exhaustively list comparisons with exponential distributions in the text, the high goodness-of-fit justifies the current choice for the simulation baseline.

Regarding robustness, our main conclusions depend on degree-distribution–dependent summary quantities. While changing the distributional form can shift the numerical value of $\rho$, the qualitative threshold structure and the comparative effectiveness of intervention strategies remain consistent across the tested distributions.

We have added a remark in Section 4 to clarify this robustness.

**Q9**: The proofs of global stability for both disease-free and endemic equilibria are highly technical. Could the authors provide more intuitive explanations or biological interpretations of these stability conditions?

**A9**: We thank the reviewer for this constructive suggestion. We agree that the mathematical proofs of global stability are technical, and adding a biological interpretation helps verify the practical significance of the model.Biologically, "global stability" implies the inevitability of the ecosystem's long-term state, regardless of the initial scale of the disturbance.

For the disease-free equilibrium ($\rho<1$): The global stability implies that if the disturbance transmission rate is lower than the recovery capacity of the system, the ecosystem possesses sufficient resilience. No matter how many species are initially disturbed, the disturbance will eventually die out, and the entire food web will return to its original healthy state.

For the endemic equilibrium ($\rho>1$): The global stability implies that if the disturbance capability exceeds a certain threshold, the ecosystem will inevitably shift to a persistent disturbed state. Regardless of initial conditions, a fixed proportion of species will remain disturbed effectively permanently, representing a long-term degradation of ecosystem function.

We have added a paragraph summarizing these biological interpretations at the end of Section 2.3.

**Q10**: In Theorem 5, the proof of global stability for the endemic equilibrium relies on a complex sequence construction. Is there a more straightforward Lyapunov function or method that could be applied?

**A10**: We appreciate the reviewer’s suggestion regarding the proof technique. We agree that Lyapunov functions generally offer a more concise and elegant approach to stability analysis.However, constructing a global Lyapunov function for the endemic equilibrium in heterogeneous network-based epidemic models is mathematically challenging due to the high dimensionality of the system. While we successfully constructed a Lyapunov function to prove local stability in Theorem 4, extending this to global stability for the endemic state remains an open problem in the field for this specific model structure.The sequence construction method employed in Theorem 5, while technically involved, is a standard and rigorous mathematical tool for establishing global stability in such high-dimensional cooperative systems where a global Lyapunov function is elusive. It ensures the result relies on robust convergence properties rather than the heuristic construction of a scalar function.

To address the reviewer's comment, we have added a Remark following the proof of Theorem 5 to explain the necessity of this method.

**Q11**: The manuscript contains several typographical and formatting issues, especially in the mathematical notation (e.g., inconsistent use of brackets, alignment in equations). A thorough proofreading is recommended.

**A11**: We sincerely apologize for the typographical and formatting errors in the original manuscript. We have conducted a thorough proofreading of the entire text as suggested.

**Q12**: Some references (e.g., [37], [38], [41]) are cited in the text but do not appear in the reference list. Please ensure all citations are complete and consistent.

**A12**: We sincerely apologize for the oversight regarding the reference list. We understand that accurate referencing is crucial for the manuscript's integrity.

We have performed a thorough cross-check between in-text citations and the reference list to ensure accuracy and completeness.

**Q13**: The figures (e.g., Fig. 2, Fig. 3) are referenced but not included in the provided text. Please confirm that all figures and tables are properly labeled and referenced.

**A13**: We sincerely apologize for the inconvenience caused by the missing figures in the previous version of the manuscript, which may have resulted from a compilation error during the PDF generation.We have explicitly verified the revised manuscript to ensure that: All Figures (Fig. 1 to Fig. 9) and Tables are correctly embedded within the main text, positioned immediately after they are first referenced. Labeling and Referencing: We have cross-checked all figure captions and table headers against the in-text citations to ensure accuracy and consistency.

**Reviewer #2**:

**Q1**: The introduction should make a compelling case for why the study is useful along with a clear statement of its novelty or originality by providing relevant information and providing answers to basic questions such as:

(a) What is already known in the literature?

(b) What was done and how was it done?

**A1**: We sincerely appreciate this constructive guidance. We agree that the original introduction did not sufficiently highlight the necessity and novelty of our work. To address this, we have substantially revised the Introduction (Section 1) to clearly articulate the research context and our specific contributions:

Regarding (a) What is already known: We have synthesized the literature to show that while complex network theory and epidemic models have been successfully applied to analyze stability in various systems, their application to ecological disturbance propagation，specifically incorporating demographic dynamics like migration，remains limited.

Regarding (b) What was done and how: We have explicitly stated our methodology: modeling ecological disturbance as an SIRS epidemic process on a complex network. Crucially, we distinguish our work by incorporating proportional migration and conducting a comparative analysis of three specific conservation strategies using both theoretical derivation and empirical simulation on the Otago pine forest food web.

We have rewritten the final part of the Introduction to clearly list the study's motivations and main contributions.

**Q2**: The model assumes disturbance propagates with a probability ρ along food web edges. Given that ecological interactions vary in strength and type (e.g., strong/weak predation, competition), how does the assumption of a uniform propagation probability $\beta$ impact the model’s biological realism? Could the model be extended to include weighted edges based on interaction strength?

**A2**：We thank the reviewer for this insightful comment. In the current framework, we use a uniform $\beta$ as a first-order approximation that represents an average propagation propensity across trophic links. This simplifying assumption allows us to isolate the role of network connectivity/topology and to derive analytical threshold and stability results. The main limitation is that link-level heterogeneity is not explicitly represented, which may affect the quantitative value of the threshold in a system-specific application, although the qualitative threshold structure and comparative insights remain.

Yes, the model can be naturally extended to include weighted edges. Mathematically, the scalar constant $\beta$ in the dynamic equations (Eq. 2.1.1) would be replaced by a transmission matrix $B=\left[ \beta_{\mathrm{ij}} \right]$, where $\beta_{\mathrm{ij}}$ quantifies the specific interaction strength or energy flow between species $i$ and $j$. While this extension would make the analytical derivation of a closed-form threshold ($\rho$) significantly more complex, it is a feasible and planned direction for our future numerical simulation studies.

We have updated the Conclusion section to explicitly document this biological limitation and the proposed matrix-based extension.

**Q3**: The expression for $\rho$ (Eq. 2.2.12) is crucial. Clarify the step-by-step derivation from the next-generation matrix method or the local stability analysis of the disease-free equilibrium. The current derivation in Lemma 3 seems to appear from a self-consistency condition; a more standard epidemiological derivation would strengthen the manuscript.

**A3**：We agree with the reviewer that establishing a rigorous connection between the basic reproduction number ($\rho$) and standard epidemiological stability analysis is essential.1. Validity of Self-Consistency:The derivation in Lemma 3 employs the "self-consistency"method, which is the standard approach in complex network theory to determine the existence of a non-trivial endemic equilibrium.2. Link to Local Stability Analysis:Crucially, as requested by the reviewer, we have actually performed the Local Stability Analysis of the disease-free equilibrium in Theorem 1 (Section 2.3).We constructed the Jacobian matrix at the disease-free equilibrium.We derived the characteristic equation (Eq. 2.3.3).We proved that the condition for the eigenvalue to be positive (instability) is exactly $\beta\frac{b}{b+\mu}\frac{\left\langle k^{2} \right\rangle}{\left\langle k \right\rangle}>\mu+\gamma+\sigma$.

This inequality is mathematically identical to the condition $\rho> 1$ derived in Lemma 3, confirming that our $\rho$ is indeed the threshold determining the stability of the disease-free state.

To strengthen the manuscript as suggested, we have added a step-by-step biological breakdown of the expression for $\rho$ in Section 2.2. The formula can be interpreted as the product of four standard epidemiological components:

$$\rho=\beta\times\frac{b}{b+\mu}\times\frac{1}{\mu+\gamma+\sigma}\times\frac{\left\langle k^{2} \right\rangle}{\left\langle k \right\rangle}$$

where $\beta$ is transmissibility, $\frac{b}{b+\mu}$ is susceptible density, $\frac{1}{\mu+\gamma+\sigma}$ is infectious duration, $\frac{\left\langle k^{2} \right\rangle}{\left\langle k \right\rangle}$ is network connectivity. This decomposition aligns the network derived threshold with classical epidemiological intuition.

We have revised Remark (1) in Lemma 3 to include this biological derivation.

**Q4**: The proofs for the global stability of both the disease-free (Theorem 2) and endemic(Theorem 5) equilibria are highly technical and rely on constructing complex Lyapunov functions and sequences. For the sake of reproducibility and clarity, provide more detailed intermediate steps or intuition behind the construction of these functions, particularly in Theorem 5.

**A4**：We thank the reviewer for this suggestion. The proof of Theorem 2 relies on constructing a linear Lyapunov function ($V\left( t \right)$) that represents the "weighted potential" of the infection. When the threshold condition $\rho< 1$ is met, the derivative of this function becomes negative definite, implying that the total "energy" of the disturbance dissipates over time, forcing the system to the disease-free state.

Regarding Theorem 5, which involves complex sequence construction, the underlying intuition is the Monotone Iteration Technique. Instead of a single energy function, we construct two sequences of bounding values:

Upper Bounds ($W, X, Y, Z$): A sequence that starts high and monotonically decreases.

Lower Bounds ($w, x, y, z$): A sequence that starts low and monotonically increases.

We prove that as the iteration step $m\to\infty$, both the upper and lower bounds converge to the exact same value (the endemic equilibrium $E_{+}$). Since the actual system trajectory $I_{k}\left( t \right)$ is mathematically trapped between these shrinking bounds, it is forced ("squeezed") to converge to $E_{+}$.

To aid clarity, we have inserted a "Proof Strategy" paragraph at the beginning of the proof for Theorem 5 in Section 2.3 to explicitly outline this logic before presenting the detailed inequalities.

**Q5**: The degree distribution for the Otago food web is fitted to a power-law $P(k)=0.26/k$ (Page 28). What statistical tests were performed to justify this specific functional form over alternatives (e.g., exponential, log-normal)? Furthermore, how were the key parameters $\beta, \sigma, \delta, \mu, \gamma, b$ assigned specific numerical values for the simulations, and were these values informed by empirical data or chosen arbitrarily for illustration?

**A5**：We thank the reviewer for scrutinizing the statistical rigor and parameter selection in our simulations. In the manuscript, we utilized the coefficient of determination ($R^{2}=0.9024$) to characterize the heavy-tailed nature of the network. We acknowledge that we did not perform rigorous comparative statistical tests against alternative distributions like log-normal or exponential forms.However, it is crucial to clarify that this fitted function was used solely for descriptive purposes to highlight the structural heterogeneity of the ecosystem. The numerical simulations and stability analyses (Section 4) were conducted using the exact empirical adjacency matrix of the 85-species Otago food web, not a synthetic network generated from the fitted curve. Therefore, the validity of the specific functional form (power-law vs. alternatives) does not influence the simulation results or the derived stability conclusions.

Regarding parameterization, the numerical values of $\beta, \sigma, \delta, \mu, \gamma$ used in simulations are selected for theoretical illustration rather than direct empirical calibration to the Otago system. Each parameter corresponds to a standard ecological process, and the chosen values lie within biologically reasonable ranges reported in the literature. Importantly, our analytical results depend on parameter relationships rather than precise values. We further include a sensitivity analysis showing that qualitative outcomes and threshold behavior are robust across broad parameter ranges.

We have added a "Simulation Setup and Data Source" subsection in Section 4 to explicitly clarify these points.

**Q6**: In Figures 5–9, the results are presented for a node of degree $k=5$. How were the initial conditions $S_{k}\left( 0 \right)$, $I_{k}\left( 0 \right)$, $R_{k}(0)$ set for these simulations, especially for the endemic case ($\rho>1$)? Specify if the results are for a single node, an average over all nodes of degree $k=5$, or a density across the network.

**A6**：We verify that the results presented in Figures 5–9 represent the density of disturbed species within the class of nodes having degree $k=5$. According to the model definition in Eq. (2.1.1), the variable $I_{k}\left( t \right)$ denotes the fraction (density) of species with degree $k$ that are in the disturbed state. The simulations were performed by numerically solving the deterministic differential equation system (Eq. 2.1.1) using the empirical degree distribution $P\left( k \right)$. Therefore, the curves shown are not stochastic realizations of a single node, but rather the average density for all nodes possessing degree $k=5$.

For the simulations, we applied a uniform initial disturbance across all degree classes to seed the propagation. Specifically:$I_{k}\left( 0 \right)=0.1$ for all $k$. This implies that 10% of the species in each degree class are initially disturbed. $R_{k}\left( 0 \right)=0$ for all $k$. $S_{k}\left( 0 \right)=N_{k}\left( 0 \right)-I_{k}\left( 0 \right)-R_{k}\left( 0 \right)$ where $N_{k}\left( 0 \right)=b/\left( b+\mu\right)$ is the carrying capacity derived from the demographic parameters.

We have updated the description in Section 4 to explicitly define these variables as densities and clarify the uniform initialization method.

**Q7**: Figures 7–9 show the system’s state evolving over time for different parameters. To assess the robustness of the conclusions, have the authors performed a sensitivity analysis on the initial conditions or the network structure itself? For instance, how do the stability results change if the network is randomized while preserving the degree distribution?

**A7**：We thank the reviewer for this excellent question regarding the robustness of our findings. As elaborated in our response to Point 7, the robustness to initial conditions is guaranteed by our mathematical proofs of Global Asymptotic Stability (Theorems 2 and 5). These theorems dictate that for any positive initial disturbance ($I\left( 0 \right)>0$), the system will inevitably converge to the unique equilibrium determined by$\rho$. Therefore, sensitivity analysis on initial conditions yields identical final steady states.

The reviewer asks how results change if the network is randomized while preserving the degree distribution. According to our derivation in Eq. (2.2.12), the basic reproduction number $\rho$ is functionally dependent only on the demographic parameters and the statistical moments of the network:$\rho\propto\frac{\left\langle k^{2} \right\rangle}{\left\langle k \right\rangle}$, Randomizing the network topology while preserving the degree distribution $P\left( k \right)$ leaves the moments $\langle k \rangle$ and $\langle k^{2} \rangle$ invariant. Consequently, the value of $\rho$ and the resulting stability conditions remain theoretically unchanged. While specific local dynamics might show minor transient fluctuations due to changes in local clustering, the macroscopic stability conclusions are robust to such structural randomization.

We have added a discussion on "Structural Robustness" in Section 4 to formally address this.

**Q8**: In Section 3, three immunization strategies are compared based on their resulting $R_{0}$($\rho_{3}<\rho_{2}<\rho_{1}$). The claim that active immunization is “most effective” relies on this inequality. However, the comparison assumes the same “cost” (i.e., the same number of protected species,$\bar{\varphi}$). Provide a more explicit comparison, perhaps with a simulation, showing the final outbreak size $I(t)$ for each strategy when the same total number of species is protected.

**A8**：We completely agree with the reviewer that a rigorous comparison requires fixing the "cost" (immunization coverage) to evaluate the "benefit" (reduction in outbreak size). First, we clarify that our theoretical comparison of the basic reproduction numbers ($\rho$) in Section 3 was indeed derived under the condition of identical total immunization density ($\bar{\varphi}=\text{constant}$) for all three strategies. Mathematically, the final endemic equilibrium size $I^{*}$ is a monotonically increasing function of $\rho$ (as implied by Theorem 5).

To provide the explicit visual comparison requested, we have performed numerical simulations fixing the total fraction of protected species at $g=0.2$ (i.e., 20% of the network is immunized under each strategy). The simulation results (added/discussed in Section 4) confirm that the Active Immunization strategy results in the lowest final stable level of disturbance $I\left( t \right)$, followed by Targeted, and then Uniform. This explicitly validates that Active Immunization yields the highest protection efficiency per unit cost.

We have added a new subsection in Section 4 to discuss this fixed-cost comparison.

**Q9**: The model employs the “empty-lattice” theory to handle network dynamics (species migration/extinction). How does this assumption of immediate replacement by an identical, susceptible node affect the long-term dynamics, especially regarding the conservation of keystone species whose loss might fundamentally alter the network topology, not just a node’s state?

**A9**：We thank the reviewer for this important point. We agree that the empty-lattice assumption whereby an extinct node is immediately replaced by an identical, susceptible node is an idealization.

In our framework, this assumption is adopted to decouple state dynamics from structural evolution, thereby preserving the degree distribution and enabling analytical tractability of long-term behavior (e.g., thresholds and global stability). As a consequence, the model focuses on how disturbances propagate on a statistically stationary network and does not capture irreversible topological changes.

This limitation is particularly relevant for keystone species, whose permanent loss can rewire interactions and fundamentally alter network structure. Under the empty-lattice assumption, such effects are intentionally suppressed; thus, the model may underestimate cascading structural consequences following keystone extinctions.

We emphasize that this choice defines the scope of the current study. A natural extension is to relax immediate replacement by allowing non-identical or delayed replacement, node-type–dependent survival, or adaptive topology. These extensions would enable explicit investigation of structural collapse and resilience under keystone loss and are promising directions for future work.

We have added a distinct paragraph in the Discussion (Section 5) to clarify the distinction between "population disturbance" and "structural extinction" and to acknowledge this limitation.

**Q10**: Figure 2 is described as a topology map but is not visually present in the provided text. If included, does it provide any topological analysis (e.g., highlighting hubs, clustering coefficient, community structure)? A simple visualization of 85 nodes is often a “hairball.” How was this figure rendered to provide insight into the network’s structure that informs the dynamical results?

**A10**：We thank the reviewer for this clarification request. Figure 2 is included in the full manuscript (and Supplementary Materials); its absence in the provided text reflects a formatting issue rather than an omission.

The goal of Figure 2 is not to present an uninformative “hairball,” but to highlight structural features relevant to the dynamics. The network is rendered using a force-directed layout, with node size proportional to degree and color indicating community/module membership. Edge transparency is adjusted to reduce visual clutter. These choices allow hubs and modular structure—both relevant to disturbance propagation and immunization effects—to be visually apparent.

We have updated the Caption of Figure 2 to include these technical details regarding the rendering method and visual encoding.

**Q11**: The simulations in Figures 5 and 6 are used to validate the theoretical threshold $\rho_{c}=1$. Do the authors observe a sharp transition in the final density of disturbed species $I(\infty)$ as $\beta$ crosses the critical value $\beta_{c}$ predicted by Eq. (2.2.13)? A figure plotting $I\left( \infty\right)$ against $\beta$(or $\rho$) would provide stronger numerical evidence for the existence of this epidemic threshold.

**A11**：We verify that the system exhibits a clear phase transition at the critical value. Mathematically, the transition at the threshold $\rho=1$ corresponds to a transcritical bifurcation. As $\beta$ crosses the critical value $\beta_{c}$, the disease-free equilibrium ($I=0$) loses stability, and the endemic equilibrium ($I^{*}>0$) becomes stable and positive. This manifests as a continuous (second-order) phase transition, where the final density $I\left( \infty\right)$ departs continuously from zero.

We have followed the reviewer’s suggestion and conducted additional simulations to plot the stationary disturbed density $I\left( \infty\right)$ as a function of the propagation probability $\beta$. The resulting curve confirms that for $\beta\leq\beta_{c}$, the final density is zero. Immediately as $\beta$ exceeds $\beta_{c}$, $I\left( \infty\right)$ becomes positive and increases monotonically. The numerical bifurcation point aligns perfectly with the theoretical prediction derived from Eq. (2.2.13), providing robust validation of the analytical threshold.

We have included this discussion and the description of the bifurcation behavior in Section 4 (associated with the sensitivity analysis in Fig. 7) to strengthen the evidence.

**Q12**: Authors may look for some punctuation, typos and editing issues.

**A12**：We thank the reviewer for this comment. We have carefully proofread the entire manuscript and corrected punctuation, typographical errors, and minor language issues. We also improved clarity and consistency in terminology and notation throughout the text.

**Reviewer #3:**

**Q1**: In the abstract, the definition of " medium-sized and large-sized " in the phrase " the medium-sized and large-sized neighbor nodes" is somewhat ambiguous. Does this refer to the degree (number of connections) of the nodes, their centrality, betweenness centrality, or other measures related to biomass/size?

**A1**：We thank the reviewer for pointing out this terminological ambiguity. We clarify that in our manuscript, the term "size" in this context refers strictly to the node degree (the number of predator-prey connections), not to biological biomass or body size. The phrase "medium-sized and large-sized neighbor nodes" was intended to describe nodes with medium to high degrees. Our results indicate that the "Active Immunization" strategy is effective because, due to the topological properties of scale-free networks, the neighbors of a randomly selected node are statistically likely to be high-degree nodes (hubs). Therefore, this strategy implicitly targets these highly connected species.

To avoid confusion with biological traits, we have replaced this ambiguous phrasing with precise topological terminology (i.e., "medium- and high-degree nodes") in the revised Abstract and throughout the manuscript.

**Q2**: The introduction provides a good overview of the application of complex network theory in ecosystems and the research foundation of infectious disease models. However, it should be supplemented with a clearer statement of the core differences and innovative aspects of this study compared to existing literature. It is recommended to explicitly highlight the unique contributions of this work in terms of model assumptions, research objectives, or empirical context, to avoid ambiguity regarding its distinction from prior research.

**A2**：We sincerely appreciate the reviewer’s suggestion to sharpen the positioning of our study. We agree that explicitly distinguishing our work from existing literature is crucial.

To address this, we have substantially revised the final part of the Introduction to highlight our unique contributions across the three specific dimensions recommended by the reviewer:

1. Model Assumptions: Unlike prior studies that often treat ecosystems as closed systems, we introduce proportional migration mechanisms (immigration and emigration rates) into the SIRS framework. This allows us to capture the dynamics of open ecosystems where species flow across boundaries is significant.
2. Research Objectives: We move beyond basic stability analysis to focus on strategy optimization. Specifically, we conduct a comparative analysis of three distinct protection strategies—Uniform, Targeted, and Active Immunization—to identify the most cost-effective approach for suppressing disturbance.
3. Empirical Context: To ensure ecological realism, we validate our theoretical thresholds not just on synthetic networks, but using the empirical topology of the Otago pine forest food web.

We have rewritten the concluding paragraphs of the Introduction to clearly explicitly state these innovations.

**Q3**: It is advisable to briefly elaborate on why the food chain can be considered the core pathway for disturbance propagation (e.g., based on the logic of energy flow and resource dependence among species), as well as the rationale behind the "immunity" analogy (e.g., how protective measures equivalently reduce the probability of species being disturbed or enhance their recovery capacity). This would strengthen the theoretical foundation of the model construction.

**A3**：We are grateful for this constructive suggestion to strengthen the theoretical grounding of our model. We agree that explicitly mapping the mathematical concepts to ecological mechanisms is essential. We clarify that the food chain is the primary conduit for disturbance because it represents the fundamental structure of energy flow and resource dependency.

We further clarify the ecological counterpart to "immunity." In our model, immunizing a node is analogous to implementing anthropogenic conservation measures. These measures artificially boost a species' resilience, making it refractory to environmental stress or population fluctuations passed down from neighbors. Mathematically, this effectively removes the species from the "susceptible" pool, mirroring the concept of vaccination in epidemiology.

We have added a paragraph titled "Ecological Interpretation of Model Assumptions" in Section 2.1 to elaborate on these points.

**Q4**: While the manuscript outlines the research framework (model construction, strategy comparison, empirical validation), the introduction lacks sufficient context for the definition of key parameters. It is suggested to briefly mention the central role of core parameters (such as disturbance propagation probability, species recovery probability, and the basic reproduction number) either at the end of the introduction or in a preliminary section of the research methods, to ensure smoother logical transition to the model derivation in subsequent sections.

**A4**：We thank the reviewer for this excellent suggestion regarding the logical flow of the manuscript. We agree that introducing the conceptual meaning of the governing parameters before the formal mathematical derivation will significantly aid reader comprehension.To bridge the gap between the general introduction and the specific model formulation, we have added a paragraph at the end of Section 1 (Introduction). This new text explicitly defines the ecological roles of the core parameters:

Disturbance Propagation Probability ($\beta$): representing the intensity of instability transmission across trophic links.

Recovery Probability ($\sigma$): representing the inherent resilience of species.

Basic Reproduction Number ($\rho$): serving as the critical threshold metric.

This addition provides the necessary context for the subsequent mathematical derivation in Section 2.

**Q5**: Standardize the literature citation format throughout the manuscript to ensure academic rigor and consistency in presentation.

**A5**：We sincerely thank the reviewer for highlighting the importance of academic rigor in citation formatting. We apologize for the inconsistencies present in the previous version. To address this, we have conducted a thorough review of the entire bibliography and in-text citations.
